# NADPH Oxidase Isoforms Are Involved in Glucocorticoid-Induced Preosteoblast Apoptosis

**DOI:** 10.1155/2019/9192413

**Published:** 2019-03-27

**Authors:** Shu-Cai Bai, Qian Xu, Hui Li, Ya-Fei Qin, Li-Cheng Song, Chen-Guang Wang, Wen-Hao Cui, Zhi Zheng, De-Wen Yan, Zhi-Jun Li, Dong Li, Xin Wan, Hua-Feng Zhang

**Affiliations:** ^1^Department of Orthopedics, Tianjin Hospital, Hexi District, Tianjin, China; ^2^Tianjin University of Traditional Chinese Medicine, Nankai District, Tianjin, China; ^3^The Department of Orthopedics, Tianjin Medical University General Hospital, Heping District, Tianjin, China; ^4^Department of Endocrinology, The Shenzhen Second People's Hospital, Health Science Center, The First Affiliated Hospital of Shenzhen University, Shenzhen, China; ^5^Department of Pharmacology, Kyoto Prefectural University of Medicine, Japan; ^6^Department of Internal Medicine 5th Division, Jiangxi Cancer Hospital, Jiangxi Cancer Center, Nanchang, China

## Abstract

Oxidative stress induced by long-term glucocorticoid (GC) use weakens the repair capacity of bone tissue. Nicotinamide adenine dinucleotide phosphate, reduced form (NADPH) oxidase (NOX) is a superoxide-generating enzyme that plays an important role in regulating bone metabolism. To clarify the role of nonphagocytic NOX isoforms in osteoblast reactive oxygen species (ROS) generation and apoptosis, dexamethasone was used to establish a high-dose GC environment *in vitro*. A dose-dependent increase in intracellular ROS generation was demonstrated, which was accompanied by increased osteoblastic MC3T3-E1 cell apoptosis. Addition of the ROS inhibitor NAC (*N*-acetyl-L-cysteine) or NOX inhibitor DPI (diphenyleneiodonium) reversed this effect, indicating that NOX-derived ROS can induce osteoblast apoptosis under high-dose dexamethasone stimulation. NOX1, NOX2, and NOX4 are NOX homologs recently identified in bone tissue. To clarify the NOX isoforms that play a role in osteoblast ROS generation, *Nox1*, *Nox2*, and *Nox4* mRNA expression and NOX2 and NOX4 protein expression were analyzed. *Nox1* and *Nox4* mRNA expression was elevated in a dose-dependent manner after culture in 100 nM, 250 nM, 500 nM, or 1000 nM dexamethasone, and the increased expression of NOX1 mRNA was more significant compared with NOX4 mRNA. Small interfering RNAs (siRNAs) were used to confirm the role of NOX1 and NOX4 in ROS generation. To clarify the signaling pathway in ROS-induced osteoblast apoptosis, mitogen-activated protein kinase (MAPK) signaling molecules were analyzed. Phosphorylated ASK1 and p38 levels were significantly higher in the 1000 nM dexamethasone group, which NAC or DPI markedly attenuated. However, the total mRNA and protein levels of ASK1 and p38 between the dexamethasone group and control were not significantly different. This is related to ROS regulating the posttranslational modification of ASK1 and p38 in MC3T3-E1 cell apoptosis. Altogether, NOX1- and NOX4-derived ROS plays a pivotal role in high-dose dexamethasone-induced preosteoblast apoptosis by increasing phosphorylated ASK1 and p38 and may be an important mechanism in steroid-induced avascular necrosis of the femoral head (SANFH).

## 1. Introduction

Glucocorticoids (GCs) are clinically prescribed anti-inflammatory and immunosuppressive medicine for treating many diseases. Excessive GC usage is the most common nontraumatic cause of osteonecrosis of the femoral head [1]. Over the last decade, accumulating evidence has demonstrated the strong association between osteocyte and osteoblast apoptosis with steroid-induced avascular necrosis of the femoral head (SANFH) [2–4]. Bone is a powerful self-repairing tissue that maintains homeostasis via osteoclast resorption and osteoblast osteogenesis [5]. However, after long-term usage of high-dose GCs, cumulative and irreparable vascular compromise and osteoblast apoptosis will occur, which could affect the balance of bone metabolism [6]. The pathological mechanism of SANFH is related to apoptosis-related molecules in osteoblasts [2, 4]. However, the specific mechanism remains unclear.

NADPH (nicotinamide adenine dinucleotide phosphate, reduced form) oxidase (NOX), an important multicomponent enzyme comprising membrane-bound catalytic subunits and several cytosolic regulatory subunits, exerts a predominant role in reactive oxygen species (ROS) generation [7, 8]. Several isoforms have been identified, including NOX1–5 and dual oxidase (DUOX)1–2, which are closely associated with regulating endothelial cell function, vascular remodeling, and genetic immune disorders [9, 10]. Recently, accumulating evidence has indicated that ROS derived from NOX1, NOX2, and NOX4 is closely related to skeletal metabolism [9, 11, 12]. Physiological ROS is an important second messenger in regulating the proliferation and differentiation of bone marrow mesenchymal stem cells, osteoblasts, and osteoclasts [13–15]. However, a dose-dependent increase in pathological ROS production will directly cause oxidative injury and dysfunction of the intracellular biological molecules [16, 17]. Apoptosis, or cell death, occurs when ROS production exceeds its elimination ability [18]. In addition, there is strong evidence that mitogen-activated protein kinases (MAPKs) are closely related to ROS-induced apoptosis [19–21]. However, NOX/NADPH oxidase isoform involvement in the pathogenesis of ROS generation and the apoptosis signaling molecules in osteoblasts induced by high-dose dexamethasone (DEX) has not been fully clarified. This prompted our investigation of whether NOX1-, NOX2-, or NOX4-derived ROS can affect osteoblast apoptosis through MAPK signaling molecules in a high-dose DEX environment. We found that NOX1 and NOX4 can induce ROS generation, which mediated osteoblast apoptosis by increasing phosphorylated ASK1 and p38 in mouse osteoblastic MC3T3-E1 cells. This provides a new target for managing NOX1 and NOX4 to provide possible therapy for SANFH.

## 2. Materials and Methods

### 2.1. Cell Culture

Murine preosteoblast MC3T3-E1 cells were purchased from the Chinese Academy of Sciences Laboratory Animal Center and maintained in Dulbecco's modified Eagle's medium (DMEM) supplemented with 10% heat-inactivated fetal bovine serum (FBS), 100 U/mL penicillin, and 100 U/mL streptomycin at 37°C in 5% CO_2_ atmosphere. To establish the high-dose DEX environment, the cells were incubated with 100 nM, 250 nM, 500 nM, or 1000 nM DEX (Sigma, St. Louis, MO) for 24 h as previously reported [22, 23]. In specific experiments, the cells were pretreated with NAC (*N*-acetyl-L-cysteine, 10 mM) or DPI (diphenyleneiodonium, 10 *μ*M) for 30 min before DEX stimulation.

### 2.2. Cell Viability Assay

Cell viability was measured by a tetrazolium (MTT) assay. MC3T3-E1 cells (2 × 10^3^ cells/well) were seeded in 96-well plates overnight and then treated as described in Cell Culture. The fresh medium containing 20 *μ*L MTT stock solution (5 mg/mL, Solarbio Biotech, China) was added and incubated at 37°C. After 4 h, the medium and MTT were removed, and the MTT formazan products were extracted with dimethyl sulfoxide (DMSO). The absorbance was read at 492 nm using an enzyme-linked immunosorbent assay plate reader. Each data point is the average of results from five wells.

### 2.3. ROS Measurement

To measure the amounts of intracellular ROS in the MC3T3-E1 cells, log-phase cells were cultured for 24 h and treated as described in Cell Culture. A dichlorodihydrofluorescein diacetate (DCFH-DA) probe (Invitrogen, Carlsbad, CA) was used as recommended by the manufacturer. The cells were incubated with DCFH-DA (10 *μ*M) at 37°C for 30 min in the dark. All samples were analyzed with at least three biological replicates, and images from each replicate were obtained with a Leica DMI4000 B fluorescence microscope (Leica, Germany). The fluorescence intensity was calculated using a Gemini EM microplate reader (Thermo Fisher Scientific, Waltham, MA). The average fluorescence intensity represented the relative ROS content.

### 2.4. Apoptosis Analysis by Flow Cytometry

MC3T3-E1 cell apoptosis was detected using a fluorescein isothiocyanate- (FITC-) annexin V apoptosis detection kit (BD Pharmingen, San Diego, CA) and flow cytometry according to the manufacturer's protocol. Briefly, the cells were washed, trypsinized, and centrifuged. Then, cells were harvested and suspended in a binding buffer for annexin V-FITC and propidium iodide (PI) staining at room temperature in the dark for 15 min. The apoptotic cells were counted by flow cytometry (BD Biosciences, Franklin Lakes, NJ) and the annexin V/PI ratio reflected the apoptosis percentage.

### 2.5. Western Blotting

MC3T3-E1 cells from the experimental groups were harvested and lysed in a lysis buffer (Solarbio Biotech). Equal amounts (30–50 *μ*g/well) of protein were separated by sodium dodecyl sulfate-polyacrylamide gel electrophoresis and transferred to polyvinylidene fluoride (PVDF) membranes. The membranes were blocked with 5% nonfat powdered milk, followed by incubation with antibodies against NOX2 (1 : 1000, Abcam), NOX4 (1 : 2000, Abcam), ASK1 (1 : 1000, Cell Signaling Technology, Danvers, MA), p-ASK1 (1 : 1000, Cell Signaling Technology), p38 (1 : 1000, Cell Signaling Technology), p-p38 (1 : 1000, Cell Signaling Technology), and *β*-actin (1 : 1000, Cell Signaling Technology) overnight at 4°C and subsequently with horseradish peroxidase-conjugated secondary antibodies for 1 h at room temperature. Immunoreactive bands were visualized by chemiluminescence (Pierce ECL). The resulting autoradiograms were analyzed by densitometry. Equal loading of proteins was qualified by detecting *β*-actin levels. Quantification was performed with ImageJ software.

### 2.6. Real-Time PCR

The total RNA of treated MC3T3-E1 cells was extracted using TRIzol (Thermo Fisher Scientific) and reverse-transcribed to complementary DNA (cDNA) using a Maxima H Minus First Strand cDNA Synthesis Kit (Thermo Fisher Scientific); mRNA expression levels were detected by real-time PCR using a kit (Sangon Biotech) according to the manufacturer's protocol. Melting curves were constructed to control for primer stability, and no-template controls (NTCs) were used to confirm lack of contamination. Data were analyzed using Bio-Rad CFX Manager software, and fold changes in gene expression were calculated using the comparative threshold cycle (CT) method (2^−ΔΔCT^) (*n* = 3). The following sequence-specific primers were used: 5′-TTCCTCACTGGCTGGGATAG-3′*Nox1* (sense) and 5′-ATTGTCCCACATTGGTCTCC-3′*Nox1* (antisense), 5′-GATCTTCTTCATCGGCCTTG-3′*Nox2* (sense) and 5′-TTCCCCATTCTTCGATTTTG-3′*Nox2* (antisense), 5′-CTTGGTGAATGCCCTCAACT-3′*Nox4* (sense) and 5′-TCAGACCAGGAATGGTTGTG-3′*Nox4* (antisense), 5′-CTGCACAATCATCGAAATGG-3′*Ask1* (sense) and 5′-CGACATGGACTCTGGGATCT-3′*Ask1* (antisense), 5′-CTGGCTCGGCACACTGATGATG-3′*p38* (sense) and 5′-TCTGCTGAAGCTGGTTAATATGGTCTG-3′*p38* (antisense), and 5′-GGTTGTCTCCTGCGACTTCA-3′*Gapdh* (glyceraldehyde-3-phosphate dehydrogenase) (sense) and 5′-TGGTCCAGGGTTTCTTACTCC-3′*Gapdh* (antisense).

### 2.7. NOX1, NOX2, and NOX4 Small Interfering RNA (siRNA) Transfection

MC3T3-E1 cells were transiently transfected using Lipofectamine 2000 with 60 nM siRNA against NOX1 (Santa Cruz Biotech, sc-43940), NOX2 (Santa Cruz Biotech, sc-35504), or NOX4 (Santa Cruz Biotech, sc-41587) according to the manufacturer's instructions. After 48 h transfection, the cells were treated with 1000 nM DEX for 24 h. PCR was performed to confirm the NOX1, NOX2, and NOX4 silencing. Then, ROS production was detected using immunofluorescence as described above and osteoblast apoptosis was analyzed by TUNEL assay.

### 2.8. TUNEL Assay

For TUNEL staining, cells were rinsed with PBS and fixed with 4% paraformaldehyde for 40 min. Then, the cells were permeabilized with 0.1% Triton X-100 for 10 min on ice and incubated in TUNEL reaction mixture for 60 min at 37°C in the dark. Finally, the cells were incubated with Hoechst (Beyotime Biotechnology) at room temperature in the dark for 5 min. TUNEL-positive nuclei and the total number of nuclei were counted in at least 5 fields in three independent experiments.

### 2.9. Statistical Analysis

Each experiment was conducted at least three times. Data are expressed as the mean ± SD from *n* independent experiments. In reference to dependent variable data, a two-way ANOVA was carried out for the different concentrations (100 nM, 250 nM, 500 nM, and 1000 nM). For statistical analyses of siRNA and MAPK signaling pathway in 1000 nM DEX, comparisons were performed by one-way ANOVA with Tukey's posttest. *p* < 0.05 was considered statistically significant.

## 3. Results

### 3.1. DEX-Induced ROS Generation Regulated MC3T3-E1 Cell Viability

**Figure 1:**
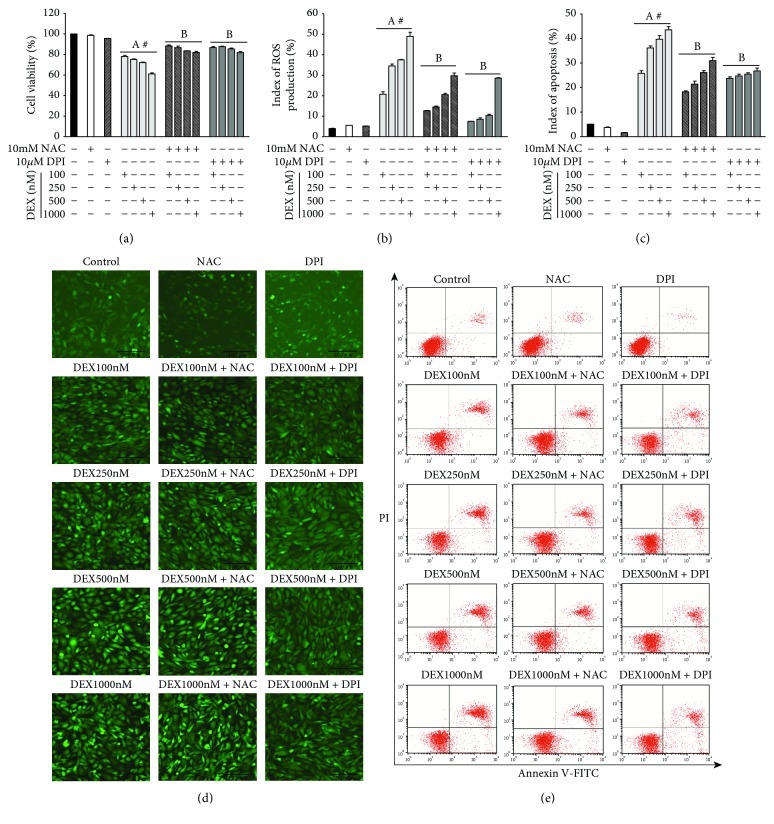
NOX-derived ROS generation plays an important role in DEX-induced apoptosis of MC3T3-E1 cells. (a) MTT assay analysis of MC3T3-E1 cell viability. (b) The relative ROS content generated was quantified by average fluorescence intensity (d). (c) The quantitative analysis of apoptotic MC3T3-E1 cells (e). (d) After 24 h exposure to DEX, the MC3T3-E1 cells were observed under inverted fluorescence microscopy (×100 magnification). (e) After 24 h exposure to DEX, annexin V-FITC/PI was used for the detection of the rate of apoptosis in MC3T3-E1 cells. ((a) *n* = 5; (b, c) *n* = 3; A, *p* < 0.01 compared with the control; B, *p* < 0.01 compared with the DEX group; #, *p* < 0.05 in the DEX group).

We used 100 nM, 250 nM, 500 nM, or 1000 nM DEX to confirm if the MC3T3-E1 cells experience oxidative injury. We used NAC (10 nM) and DPI (10 *μ*M) for 30 min to block ROS generation and NOX expression in selected groups. MTT assay showed that DEX inhibited osteoblast viability in a dose-dependent manner (Figure 1(a)). However, NAC or DPI intervention reversed the inhibition of DEX on MC3T3-E1 cells, suggesting that high-dose DEX (100–1000 nM) can inhibit MC3T3-E1 cell viability. In addition, NOX and ROS play a significant role in osteoblast viability in response to high-dose DEX.

### 3.2. NOX Was the Main Source of ROS

We further evaluated the effect of osteoblast NOX on ROS generation and the apoptotic effect in response to DEX *in vitro*. MC3T3-E1 cells were incubated for 24 h incubation with DEX before immunofluorescence assay and annexin V-PI assay were performed. DEX increased ROS generation at 100 nM (5.2-fold), 250 nm (8.7-fold), 500 nM (9.5-fold), and 1000 nM (12.4-fold) compared to the control (Figure 1(b)), which was significantly different in a dose-dependent manner. In addition, the rate of apoptosis in the DEX group was significantly increased following incubation with 100 nM (5.1-fold), 250 nm (7.2-fold), 500 nM (7.9-fold), and 1000 nM DEX (8.6-fold) compared to the control (Figure 1(c)). However, NAC or DPI significantly reduced ROS levels and DEX-induced apoptosis, confirming that NOX-derived ROS plays an important role in apoptosis in response to high-dose DEX in MC3T3-E1 cells.

### 3.3. NOX1 and NOX4 Mediated ROS Production and Osteoblast Apoptosis in a High-Dose DEX Environment

**Figure 2:**
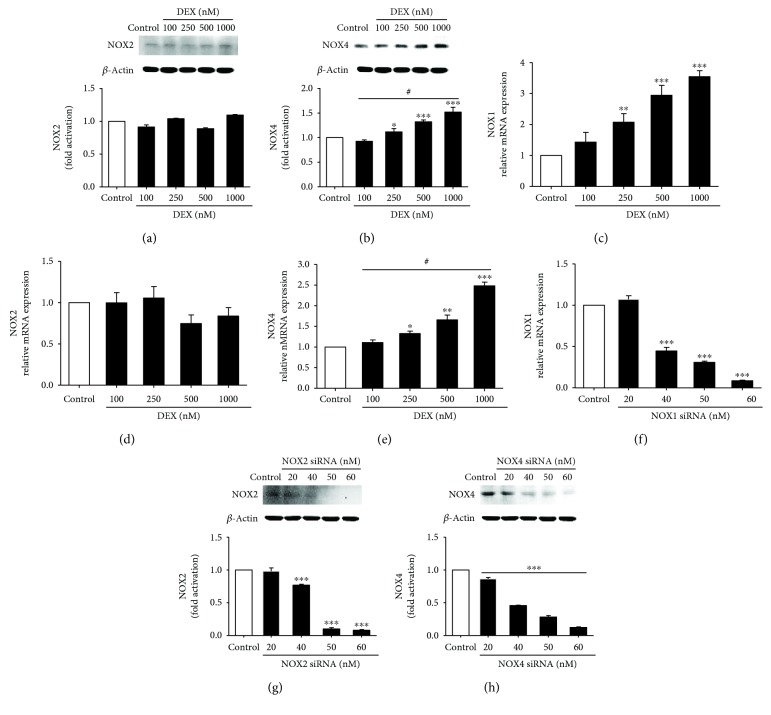
DEX increases NOX1 and NOX4 expression in MC3T3-E1 cells, and the NOX1, NOX2, and NOX4 siRNA efficiencies were detected. (a) Representative immunoblots and densitometric analyses of NOX2. (b) Representative immunoblots and densitometric analyses of NOX4. (c) DEX increased *Nox1* mRNA expression. (d) *Nox2* mRNA levels induced by DEX. (e) DEX increased *Nox4* mRNA expression. (f) RT-PCR detection of *Nox1* mRNA in MC3T3-E1 cells transfected with NOX1 siRNA for 48 h. (g) Western blot detection of NOX2 protein expression following 48 h NOX2 siRNA transfection. (h) Western blot detection of NOX4 protein expression following 48 h NOX4 siRNA transfection (*n* = 3; ^∗^*p* < 0.05 compared with the control; ^∗∗^*p* < 0.01 compared with the control; ^∗∗∗^*p* < 0.001 compared with the control; ^#^*p* < 0.05 in the DEX group).

To assess the NOX isoforms involved in ROS regeneration and apoptotic effect in osteoblasts in response to high-dose DEX, the mRNA and protein expression levels of NOX2 and NOX4 were detected by real-time PCR and western blotting, respectively, and *Nox1* mRNA levels were detected using real-time PCR. We analyzed the NOX2 and NOX4 protein expression levels in response to 100–1000 nM DEX. The DEX group had significantly higher *Nox1* mRNA levels at 100 nM (1.4-fold), 250 nm (2.1-fold), 500 nM (2.9-fold), and 1000 nM DEX (3.5-fold) than the control group (Figure 2(c)). NOX4 protein expression was significantly increased at 250 nm (1.1-fold), 500 nM (1.3-fold), and 1000 nM DEX (1.5-fold) compared to the control group (Figure 2(b)), and there was a similar increase in *Nox4* mRNA expression in the DEX group (Figure 2(e)). However, there was no significant expression of NOX2 in the DEX group (Figures 2(a) and 2(d)). In contrast, NOX1 expression was markedly higher than NOX4 expression in all specimens from the DEX group. Therefore, high-dose DEX induces elevated NOX1 and NOX4 expression in MC3T3-E1 cells in a dose-dependent manner, and the contribution of elevated NOX1 was more significant.

**Figure 3:**
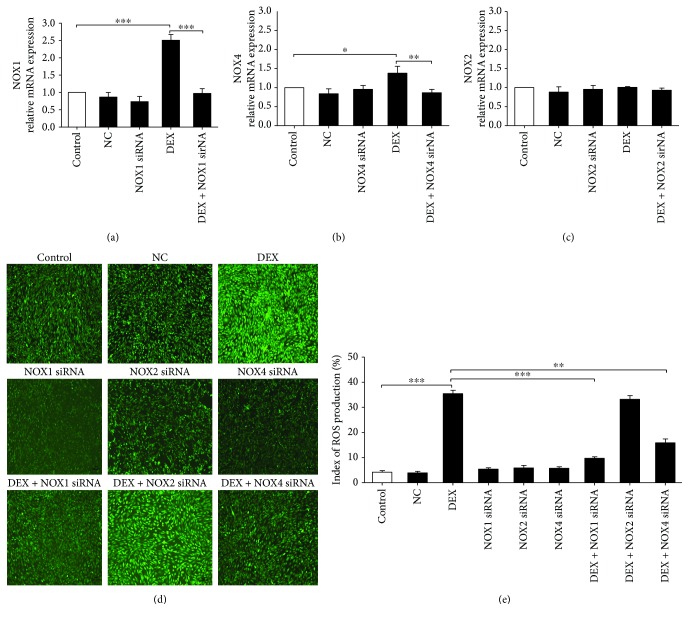
NOX1 and NOX4 are involved in DEX-induced ROS production. (a–c) PCR confirmation of NOX1 (a), NOX4 (b), and NOX2 (c) silencing in MC3T3-E1 cells transiently transfected with siRNA (60 nM) for 48 h. (d) DCFH-DA probe detection of intracellular ROS in siRNA-transfected osteoblasts treated with 1000 nM DEX for 24 h. (e) Quantitative analysis of DCF fluorescence intensity in (d) (*n* = 3; ^∗^*p* < 0.05; ^∗∗^*p* < 0.01; ^∗∗∗^*p* < 0.001).

**Figure 4:**
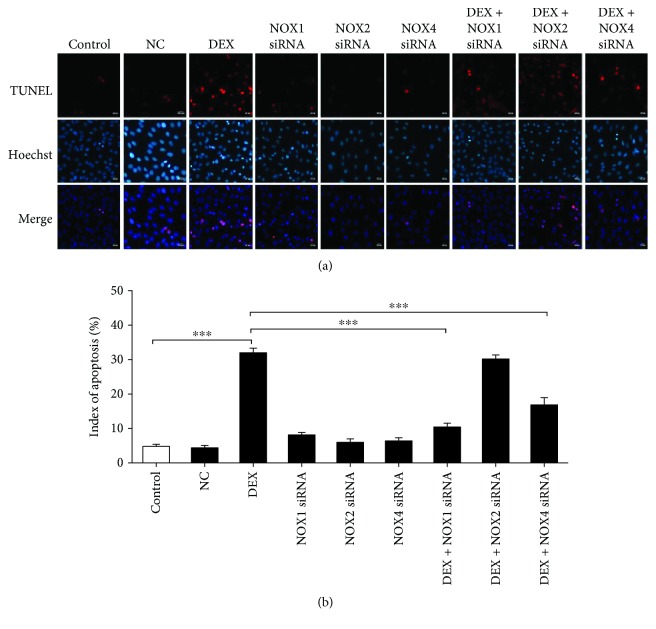
NOX1 and NOX4 are involved in DEX-induced osteoblast apoptosis. (a) TUNEL assay detection of apoptosis in siRNA-transfected osteoblasts treated with 1000 nM DEX for 24 h. (b) Quantitative analysis of osteoblast apoptosis rate in (a) (*n* = 5; ^∗∗∗^*p* < 0.001).

NOX1, NOX2, and NOX4 siRNAs were used to confirm the role of specific NOX in ROS generation and osteoblast apoptosis. After siRNA transfection, the osteoblasts were cultured for 24 h with 1000 nM DEX before ROS production was detected by immunofluorescence; osteoblast apoptosis was observed by TUNEL assay. NOX1, NOX2, and NOX4 expression was silenced in the presence of 60 nM siRNA for 48 h (Figures 2(f)–2(h); Figures 3(a)–3(c)). NOX1 or NOX4 knockdown significantly decreased the fluorescence intensity and osteoblast apoptosis (Figures 3(d) and 3(e); Figures 4(a) and 4(b)). However, such effects were not observed following NOX2 knockdown. Taken together, the results confirm that NOX1 and NOX4 silencing is associated with significantly reduced ROS generation and apoptosis in MC3T3-E1 cells. In addition, the role of NOX1 was more significant.

### 3.4. ROS Mediated ASK1 and p38 Phosphorylation in DEX-Induced Osteoblast Apoptosis

**Figure 5:**
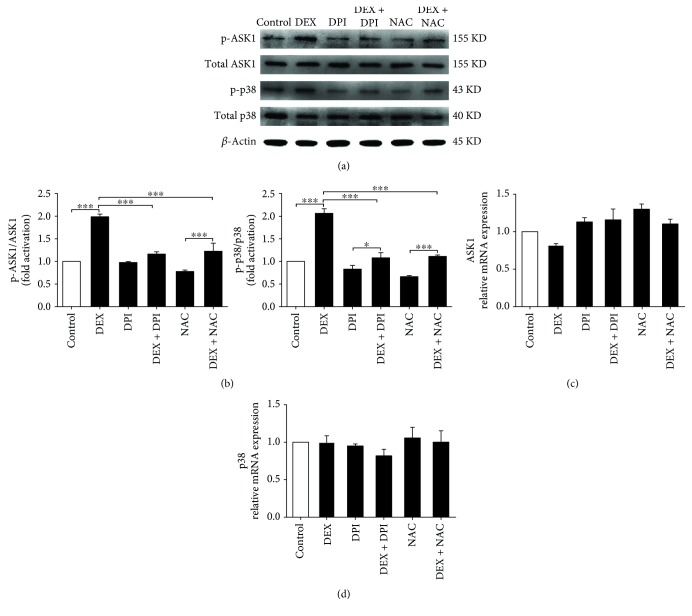
NOX-derived ROS generation regulates the ASK1 and p38 phosphorylation in osteoblast apoptosis. (a) Representative immunoblots of p-ASK1, ASK1, p-p38, and p38 in osteoblast induced by 1000 nM DEX. (b) Representative densitometric analyses of p-ASK1/ASK1 and p-p38/p38. (c) RT-PCR detection of *ASK1* mRNA in MC3T3-E1 cells. (d) RT-PCR detection of p38 mRNA in MC3T3-E1 cells (*n* = 3; ^∗^*p* < 0.05; ^∗∗∗^*p* < 0.001).

MAPK signaling is considered an important signaling pathway closely associated with apoptosis [24, 25]. To clarify the levels of apoptosis-related signaling molecules induced by ROS in osteoblasts, western blotting and reverse transcription- (RT-) PCR were used to detect ASK1, p-ASK1, p38, and p-p38 expression following stimulation with 1000 nM DEX. The total mRNA and protein levels of ASK1 and p38 in the DEX group have no significant difference compared with the control group. (Figures 5(c) and 5(d)). However, in contrast with the control group, the level of p-ASK1 and p-p38 in the DEX group was significantly higher. In addition, NAC or DPI markedly attenuated phosphorylated ASK1 and p38 levels (Figures 5(a) and 5(b)). This is probably because ROS regulates the posttranslational modification of ASK1 and p38 in MC3T3-E1 cell apoptosis.

## 4. Discussion

We report osteoblastic MC3T3-E1 cell apoptosis induced by oxidative injury in a high-dose DEX environment. Further investigation demonstrated the role of NOX1- and NOX4-derived ROS in high-dose DEX-induced osteoblastic MC3T3-E1 cell apoptosis *in vitro*. The principal findings were as follows: (1) high-dose DEX induces a marked increase in NOX1-derived and NOX4-derived pathological ROS generation in osteoblasts, and the effect of NOX1 is more significant compared with NOX4; (2) increased ROS production can lead to osteoblast apoptosis in a dose-dependent manner; and (3) in osteoblast apoptosis, pathological ROS is an important molecule for stimulating ASK1 and p38 phosphorylation by regulating posttranslational modification. In short, NOX1 and NOX4 are probably important targets in contributing to bone repair in early SANFH.

Recently, increased osteoblast and osteocyte apoptosis was demonstrated in steroid-induced avascular necrosis of the femoral head [2–4]. Since Weinstein and colleagues demonstrated osteocyte apoptosis in GC-induced osteonecrosis of the hip [26], an increasing number of studies have indicated that oxidative injury accompanied by osteoblast apoptosis plays an important role in SANFH [3]. *In vitro* and *in vivo* studies have also reported that excessive GCs induce oxidative injury and cause osteoblast apoptosis [6, 27]. These findings may indicate an important oxidative stress-induced mechanism involved in osteoblast apoptosis that could lead to SANFH.

ROS is the master molecule that can lead to oxidative injury [17, 28]. As important sources of intracellular ROS, NOX1, NOX2, and NOX4 can regulate bone immune microenvironment processes [9, 29–31]. Mandal and colleagues reported that physiological NOX4 promotes bone mesenchymal stem cell differentiation into osteoblastic cells [13]. In addition, NOX1, NOX2, and NOX4 play a vital role in osteoblast secretion of colony-stimulating factor (CSF-1) and tumor necrosis factor superfamily member 11 (RANKL), which leads to bone loss by regulating osteoclast proliferation and differentiation [32]. However, pathological factors such as alcohol and aberrant mechanosignaling can lead to NOX overexpression, causing excessive intracellular ROS generation [33, 34]. Then, apoptosis is activated, which affects the self-repair and regeneration of bone tissue [35, 36]. However, the relationship between NOX and GC-induced osteoblast apoptosis is not fully understood. In the present study, a concentration gradient of high-dose DEX was used to investigate the effect on osteoblast apoptosis. High-dose DEX inhibited MC3T3-E1 cell viability effectively in a dose-dependent manner. However, DPI or NAC reversed this inhibitory effect, indicating that NOX is an important source of ROS.

We investigated NOX1, NOX2, and NOX4 mRNA and protein expression to clarify the NOX isoforms in DEX-induced osteoblast ROS generation. NOX1 and NOX4 played a significant role in ROS production. Compared to NOX4, the contribution of elevated NOX1 was more pivotal in the osteoblastic MC3T3-E1 cells. Subsequently, we targeted NOX1, NOX2, and NOX4 individually with siRNA to silence mRNA and protein expression. The results suggested that siRNA silencing of NOX1 or NOX4 suppressed ROS production significantly in osteoblasts induced by 1000 nM DEX, especially the role of NOX1, which is in accordance with the findings of previous studies. Unfortunately, we have not found a suitable NOX1 antibody yet. Therefore, we only detected the protein expression of NOX2 and NOX4.

Previous studies have indicated that nonmacrophage NOX can produce low levels of intracellular ROS, which is recognized as a second messenger for mediating stress-sensitive signal transduction for maintaining cellular physiological functions [37, 38]. However, excessive ROS generation results in intracellular organelle damage and apoptosis-related signaling pathway activation [39, 40]. Recent research has reported that osteoblasts can attenuate intracellular oxidative injury by autophagy to maintain cell structure and function [41–43]. However, increased ROS production in osteoblasts will exceed the osteoblast antioxidant capacity and accelerate apoptosis [44]. MAPKs are widely recognized as major cell signaling molecules in ROS-induced apoptosis [19–21]. However, the specific molecular mechanisms in osteoblasts have not been fully elucidated. In our experiment, there was no significant increase in the total mRNA and protein levels of ASK1 and p38 during ROS-induced apoptosis, suggesting that ASK1 and p38 phosphorylation operated the process of ROS-induced osteoblast apoptosis. ASK1 (MAPK1) is an apoptosis-related protein switch that activates p38 (MAPK2) by adding a phosphate group to them via phosphorylation [45]. The reversible process of protein kinase phosphorylation plays an important role in activating the downstream molecules in osteoblast apoptosis. In addition, DPI or NAC could reduce the ASK1 and p38 phosphorylation effectively, which is in accordance with previous study findings.

In summary, we demonstrate that NOX1- and NOX4-derived ROS is involved in high-dose DEX-induced osteoblastic MC3T3-E1 cell apoptosis by increasing phosphorylation of the ASK1-p38 signaling pathway. Therefore, early targeted intervention of NOX1 and NOX4 may provide viable means of treating SANFH in the future.

## Data Availability

The data used to support the findings of this study are included within the article.
